# Evaluation of intra-articular injection of collagen-elastin hydrogel microparticles for managing osteoarthritis-associated elbow pain in dogs: a double-blind, positive-controlled clinical trial

**DOI:** 10.3389/fvets.2026.1742766

**Published:** 2026-02-11

**Authors:** Gabriella Castro, Lindsay Hochman Elam, Felix Michael Duerr

**Affiliations:** Department of Clinical Sciences, Colorado State University, Fort Collins, CO, United States

**Keywords:** canine elbow dysplasia, collagen-elastin hydrogel microparticles, injection, intra-articular, orthopedics, osteoarthritis

## Abstract

This double-blind, positive-controlled clinical trial was designed to evaluate the tolerance and efficacy of a commercially available collagen-elastin hydrogel microparticle (CEHM) injectate. Thirty-five client-owned dogs with naturally occurring bilateral elbow osteoarthritis (OA) were randomized into two study groups and received a single intra-articular injection in both elbows: Triamcinolone (TA) + CEHM or TA only. Eighteen dogs were treated with TA + CEHM; 17 were treated with TA only. All patients were required to be on a consistent regimen of nonsteroidal anti-inflammatory drugs (NSAIDs) at the time of enrollment and were instructed to discontinue them before injection. The patients were evaluated at seven time points over a one-year period. Outcome measures included veterinary assessment, objective gait analysis (OGA), accelerometry, and clinical metrology instruments (Canine Brief Pain Inventory and Client Specific Outcome Measures). Individual patient success was predefined based on previous literature as demonstrating specific improvements in four out of five of the following outcome measures: Veterinary assessment, OGA, accelerometry, Canine Brief Pain Inventory Pain Interference Score, and Canine Brief Pain Inventory Pain Severity Score. Thirty-three patients were included for at least partial data analysis. Adverse effects associated with TA + CEHM included three mild and two moderate cases of transient soreness. Two mild cases of transient soreness were reported in the TA group. All adverse events resolved without treatment. There was insufficient evidence to conclude a difference between groups for any of the response variables. As expected, clinical metrology instruments improved significantly in both groups. In total, 82% (14/17) of dogs in the TA + CEHM group resumed NSAID treatment, compared to 56% (9/16) of dogs in the TA group. The proportion of dogs achieving individual patient success was low and ranged from 0 to 29% across the study time points. Given the lack of difference between groups, further research, with larger sample sizes, is needed to justify the use of these products in dogs for the treatment of elbow OA.

## Introduction

Canine elbow osteoarthritis (OA) is a prevalent and debilitating condition characterized by progressive articular cartilage degradation, soft tissue pathology, subsequent pain, and functional impairment, significantly impacting the quality of life of affected dogs (1). A recent study suggests that the elbow is the most common appendicular joint to be affected by OA in dogs, accounting for approximately 18% of appendicular OA cases in dogs between 8 months and 4 years old (2). Wright et al. reported that 38% of dogs over 1 year of age that were previously undiagnosed with OA showed signs of OA when their owners responded positively to at least one question indicating a mobility disorder/pain (3). Roitner et al. found that OA prevalence in dogs older than 8 years was highest in the elbows at 57.4%, compared to the shoulders (39.2%), hips (25.9%), and stifles (36.4%) (4).

Successful treatment of elbow dysplasia is difficult due to the complexity of the joint and the disease itself. Current therapies, including non-steroidal anti-inflammatory drugs (NSAIDs), physical therapy, nutraceuticals, and surgical intervention, aim to alleviate clinical signs and, ideally, modify the disease process. Many surgical options exist with varying levels of evidence for alleviating clinical signs, including subtotal coronoidectomy, ununited anconeal process stabilization/removal, ulnar osteotomy, biceps ulnar release procedure, and total joint replacement (5–7). Surgical intervention for coronoid disease is somewhat controversial, given that recent research has failed to identify a clear superiority over medical management (8, 9).

Intra-articular corticosteroid and orthobiologic injections are used to reduce pain and modulate inflammation, aiming to provide temporary relief from clinical signs (10). To date, a successful treatment for dogs affected by OA of the elbow joint is lacking. Corticosteroids have historically been used as an intra-articular treatment for OA, however, repeat injections have been linked to cartilage degradation in people making them less suitable for long term use (11). In recent years, there has been growing interest in regenerative medicine strategies for treating canine OA. One novel approach involves the use of injectable hydrogels, which can create scaffolding for joint cushioning and drug delivery (when combined with other injectable drugs) (12).

Recently, an injectable product made of collagen-elastin hydrogel microparticles (CEHM) has become commercially available to veterinarians in the United States. According to the manufacturer, this novel product is a gelatinous, particulate substance composed of naturally derived proteins and carbohydrates from bovine and porcine tissues. The particles are made of type I collagen, elastin, and heparin, which have been suggested to self-assemble to form a novel biomaterial that acts as tissue scaffolding based on its similarity to naturally occurring extracellular matrix. This product is composed of materials that are “generally regarded as safe” by the FDA (13). According to the manufacturer, immediately after injection, CEHM may integrate with the synovial fluid to enhance lubrication and viscosity. Additionally, CEHM has been suggested by the manufacturer to integrate into joint tissues to create a joint scaffold that mimics the extracellular matrix to provide structural support for cell growth and tissue regeneration. Finally, the CEHM-created scaffold has been suggested to facilitate drug delivery when combined with other injectable products. A 2024 article by Lin et al. suggests that hydrogels may fill defects to provide a tissue scaffold allowing for the capture and release of cells, drugs, and other bioactive molecules (14).

Other hydrogels are currently being used in equine medicine (e.g., 2.5% polyacrylamide hydrogel; PAAG). PAAG is a particulate, homogeneous substance that forms cross-links, allowing the gel to maintain its molecular stability (15). Several studies have been performed evaluating this product’s efficacy and safety in horses (16, 17). A double-blind, positive-controlled study comparing PAAG to TA or sodium hyaluronate (HA) for the management of middle carpal joint lameness in racing thoroughbreds showed significant lameness improvement when treated with PAAG compared to TA and HA at 6 and 12 weeks based on blinded examinations (18).

Several safety and efficacy trials conducted by the manufacturer have evaluated the intra-articular injection of the CEHM product tested in this study in dogs. These trials reported no adverse events and confirmed the biocompatible nature of this product in dogs and cats (19–22). A safety trial conducted by the manufacturer using 20 healthy beagles showed no difference in lameness, joint swelling, or pain between joints injected with CEHM and joints injected with saline (22). Preliminary data from a trial performed in 40 dogs with unilateral cranial cruciate ligament disease suggested significant improvement in all outcomes (Glasgow Composite Pain Score, visual lameness score, and Liverpool Osteoarthritis in Dogs survey) (19). Additional preliminary data from a trial in dogs with radiographic evidence of bilateral hip OA and pain found that 7/9 dogs had a greater than 3-point reduction in total Canine Brief Pain Inventory (CBPI) score and improvement in hip extension on goniometry. All nine patients showed improvement in the visual lameness score and quality-of-life assessment (20). However, to date, no placebo-controlled, blinded studies have investigated the efficacy of this novel treatment.

In this double-blind, positive-controlled study, the researchers aimed to evaluate the tolerance and efficacy of injecting CEHM into the elbow joints of dogs with naturally occurring OA.

## Materials and methods

The study design was approved by the Clinical Review Board of Colorado State University (IACUC: #1159), and owner consent was obtained prior to enrollment. Client-owned dogs of any breed or sex presenting to the Colorado State University Veterinary Teaching Hospital were considered for enrollment. Inclusion criteria included dogs ≥10 kg with a body condition score (BCS) of 4–8/9, as measured using a previously published body condition scale (23). The patients had to be generally healthy, without evidence of progressive systemic or neurologic disease, and demonstrate clearly identifiable weight-bearing lameness (grade 1 or higher on the Subjective Orthopedic Score [SOS]) (24). Lameness had to be clinically attributable to bilateral OA of the elbow joints. Patients were required to have a score of ≥ 2 on the CBPI average pain severity score (PSS), the pain interference score (PIS), and the Canine Osteoarthritis Staging Tool (COAST, excluding radiograph scoring). Enrollees had to be accustomed to always wearing a collar to facilitate accelerometry. A consistent OA treatment protocol (supplements, medications, etc.), including consistent NSAID administration, was required for at least 4 weeks prior to enrollment.

Dogs affected by brachycephalic airway syndrome or any systemic illness that would have prevented safe sedation of the patient were excluded. Also excluded were patients with unstable cruciate disease and those who had received elbow surgery, joint injections in either elbow, or who had septic arthritis in either elbow within 6 months prior to enrollment.

At enrollment, each patient received a baseline orthopedic examination, blood work (complete blood count and chemistry panel), an objective gait analysis (OGA), clinical metrology instruments (CMIs; CBPI, Client-Specific Outcome Measures [CSOM]), and COAST (using CBPI, excluding a radiographic evaluation). The percent body weight distribution (%BWD) of the more severely affected limb had to be below the normal range (defined as <25% BWD) (25) and/or >10% less than the contralateral thoracic limb, if not below the normal range. Patients were required to have a diagnosis of bilateral elbow OA via radiographs or computed tomography (CT) within the preceding six months. Owners were informed that changes to the patient’s OA management regimen (i.e., changing medications, changing supplements, diet changes, or other new treatments) should be avoided unless required for pain control, and must be reported to the investigators.

At Visit 2, patients were assessed for disease progression to confirm a “stable disease state”, defined as: the affected limb continuing to fulfill the inclusion criteria for OGA, and the PSS and PIS scores not changing by more than 1 and 2 points, respectively.

### Diagnostic joint anesthesia (DJA)

To increase confidence in the localization of the patient’s pain, patients were required to undergo DJA prior to study enrollment. DJA was performed at Visit 2, 15 days after baseline data was obtained, if stable disease criteria were met. DJA was performed on only the elbow of the more clinically affected limb, as previously described (26) with OGA used instead of subjective gait analysis. Sedation was administered to all patients using dexmedetomidine (2.5–10 mcg/kg) and butorphanol (0–0.2 mg/kg), as needed for adequate sedation. To qualify for study enrollment, patients had to display an improvement of ≥ 5 %BWD on the injected limb compared to the pre-DJA value. The following formula was used to calculate this value:


$$\frac{\%\mathrm{BWD}(\text{post block})-\%\mathrm{BWD}(\mathrm{pre}\;\text{block})}{\%\mathrm{BWD}(\mathrm{pre}\;\text{block})}\times 100=\%\text{improvement}.$$

### OA scoring

Imaging (CT or radiographs) was evaluated in a blinded fashion by a board-certified veterinarian (FD) to establish a subjective OA score. The more clinically affected elbow was scored using a visual analog scale (from 0 to 100) using the following features to determine a global OA score: sclerosis, lysis, osteophytosis, and enthesophytosis. Zero was defined as a normal elbow without evidence of OA. One hundred was defined as end-stage OA (i.e., it could not be any worse). Scoring was performed only at enrollment to define the degree of OA present, and it was not repeated at any point during the study.

### Treatment groups

Dogs were randomly assigned to one of two treatment groups: TA (Triamcinolone acetate injectable suspension—Kenalog-10®, 10 mg/mL; TA, Princeton, NJ, USA) or TA + CEHM (CEHM provided by the study sponsor [PetVivo, Inc.] Minneapolis, MN, USA). Blocked randomization (group = 8 dogs) was used to achieve a 1:1 treatment group allocation (Random.org; Dublin, Ireland). Injection of TA or TA + CEHM in the bilateral elbows occurred at Visit 3 according to the doses listed in Table 1. The TA dose (but not the CEHM dose) was adjusted for lean body weight as needed (i.e., 10% was subtracted from the dose for each point above a 5/9 BCS).

**Table 1:** Study drug dosing.

Study dosing		
Dog weight (in kg)	CEHM dosing (mL/joint)	TA dosing (mg/joint)
10–25	1.5	2
25–40	2	3
40–55	2.5	4
>55	3	5

All clinicians performing SOS scoring and the owners were blinded to the treatment groups. Owners were advised to discontinue NSAIDs 5 days before injection. The injections were performed by one of two clinicians (LE or FD) not involved in the SOS scoring. The injections were performed using an aseptic technique with joint fluid aspiration to confirm intra-articular needle placement. The syringe was wrapped in tape to blind the contents to other staff members (since the injection appearance and volume differed by treatment groups). For ethical reasons, owners were instructed that they could resume NSAIDs after the injection if needed for pain control. Three days post injection, owners were sent a survey with questions about adverse events/their dog’s response to the injection.

**Figure 1:**
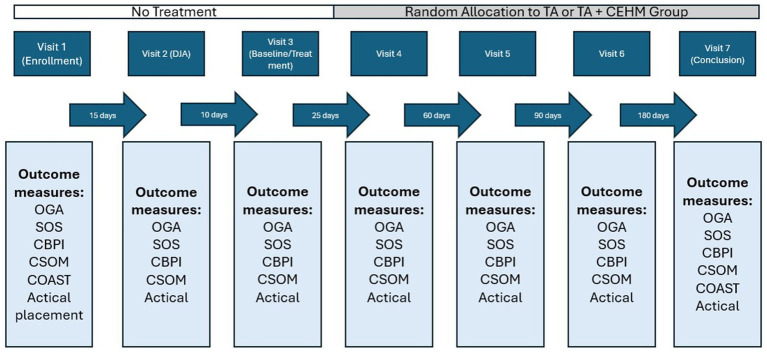
Study timeline and outcome measures.

Patients were evaluated at seven time points over a one-year period. A study timeline is provided in Figure 1. Owners were instructed that if their dog required further treatment during or after the study, they could receive the CEHM product if their pet was originally in the TA group. If owners elected to unblind the study for this reason, all subsequent data were excluded.

### Outcome measures

#### CMIs/COAST

CMIs, including the CBPI and CSOM, were completed by the owners at each visit. The COAST was completed by owners and clinicians at enrollment and at the final study visit. Radiographs were excluded from the COAST evaluation and were instead evaluated separately so as not to dictate the COAST score. The CBPI was used as the CMI for the COAST. At enrollment, research staff discussed the CMIs with the owners to answer any questions regarding the CMIs. Owners were provided with a list of suggested activities and behaviors for the CSOM (24). Owners were allowed to choose outcomes not listed after discussion with the research staff. Due to the ability to choose positive and negative behaviors, the values were adjusted from negative to positive for statistical analysis so that all larger numbers represented favorable outcomes.

#### Veterinary assessments

A complete physical and orthopedic examination was performed by a veterinarian at every visit, and SOS was recorded as previously reported (24). SOS scoring included lameness, disability, weight bearing, and pain on joint manipulation. The most affected elbow (determined at enrollment) was used for SOS scoring. Weight, vital parameters, and BCS were also recorded at each visit.

#### Accelerometry

An accelerometry collar (Actical; Respironics Mini Mitter Division, Bend, OR, USA) was worn by all patients throughout the study period (27). The accelerometer was attached to a dedicated collar, and owners were instructed not to use this collar for leash walking. The epoch length was set to 60 s, and monitoring was continuous throughout the study period. Owners were instructed to leave the collar on at all times. Data were recorded, and collars were charged at every study visit.

#### OGA

OGA was performed at each visit using a pressure-sensitive walkway (PSW) (6-Tile High Resolution Strideway System, Tekscan Inc., South Boston, MA, USA) (25). Data were collected using a previously described protocol (28). %BWD of the most affected limb (determined at enrollment) was used for data analysis.

#### Individual patient success

Predefined success criteria were used for individual patient outcome assessment. Individual patient success was defined as meeting four of five of the following outlined success criteria:

1. >3.5% increase in %BWD in the most affected limb when compared to baseline (29).
2. Reduction ≥ 1 in PSS (30).
3. Reduction ≥ 2 in PIS (30).
4. Reduction > 2 SOS-scoring.
5. Increase in activity, defined as at least one of the following:

- >20% increase in total weekly activity counts (31).
- >20% increase in time spent in activity category “moderate” (27).
- >20% increase in time spent in activity category “vigorous” (27).

Baseline data (Visit 3) was compared to each time point throughout the trial.

#### Post-injection survey

Three days after the injection, a survey (Table 2) was emailed to all owners. The survey instructed owners “please answer the following questions based on your dog’s mobility 3 days after elbow injections compared to mobility before injections” [sic].

**Table 2:** Post-injection survey.

Question	Response				
How are they walking today?	Much worse	A little bit worse	Same	A little bit better	Much better
How is the limping?	Limping less	No change in limping	Limping more		
How would you rate their difficulty rising?	Less difficult to rise	No change in rising ability	More difficult to rise		
Do you notice any heat or swelling in either elbow?	Yes	No	Not sure		
Is your dog non-weight bearing in either front limb (i.e. completely lifting up the leg all the time?	Yes	No	Not sure		
Any other comments/observations:	Free response				

This survey was sent to owners 3 days after injection with TA + CEHM or TA.

If owners responded negatively to any of the questions, they were contacted by a clinician, and a recheck was scheduled if warranted. The diagnosis of transient soreness was recorded if owners indicated “more difficulty rising,” and/or “limping more.” Responses to the question “How is he/she walking today?” were used to differentiate between mild and moderate transient soreness, with “a little worse” coded as mild and “much worse” as moderate. If owners reported noticing heat, swelling, or non-weight-bearing lameness, a recheck visit was encouraged.

### Statistical analysis

Baseline data from previous internal research (*n* = 23 dogs) with similar inclusion criteria were used to determine the sample size. The sample size calculation was performed using SAS Proc Power (SAS 9.4, SAS Institute Inc., Cary, NC, USA) corresponding to a between-groups comparison with an alpha = 0.05. The CBPI was used as the primary outcome measure. Based on this analysis, for the CBPI PSS with a conjectured standard deviation of 1.68 and a difference between means of 2, *n* = 13 dogs per group were required to achieve 80% power. For CBPI PIS with a conjectured standard deviation of 2.10 and a difference between means of 2, *n* = 19 dogs per group were required to achieve 80% power. Given expected attrition over the study period, a sample size of *n* = 30 dogs per group was determined to be appropriate for the primary outcome measure, the CBPI.

All statistical analyses were conducted using R software (The R Foundation, University of Auckland). Data obtained from Visit 3 (before injection) were used as the baseline, except for COAST, where data from Visit 1 were used. Summary statistics—including the mean, median, standard deviation, minimum, first quartile (Q1), third quartile (Q3), and maximum—were calculated for all numerical response variables and arranged by treatment group and event (timepoint). For all mixed models, “dog” was included as a random effect to account for repeated measures. For each mixed model, tests of main effects and interactions were calculated. Pairwise comparisons between groups were only considered for those variables showing evidence of a group or group*event interaction. Dunnett-adjusted comparisons across time were considered for those variables showing evidence of an event main effect. Linear mixed models were used to analyze the majority of numerical variables, %BWD, SOS, CSOM Activity Mean (CSOM ACT), CSOM Behavior Mean (CSOM BEHAV), CBPI PSS, and CBPI PIS, using pre-injection events. Group comparisons were performed at baseline. A t-test was run for comparison at baseline between groups for %BWD, SOS, CSOM (ACT and BEHAV), CBPI (PIS and PSS), OA score, age, and weight. A Wilcoxon rank-sum test was run for comparisons at baseline of CBPI quality of life (QOL) and COAST. Linear mixed models were fit separately for the numerical response variables (except for the OA score and the %improvement on DJA). For these mixed models, group (TA + CEHM or TA), event, and the interaction between group and event were included as fixed effects. NSAIDs were included as a covariate in the model. Dunnett-adjusted comparisons were used to compare each event against the baseline. The CBPI QOL and COAST scores were analyzed as ordinal outcomes (scored 1–5) and were not normally distributed. CBPI QOL scores were evaluated using Wilcoxon rank-sum tests at each event to compare treatment groups, with Bonferroni adjustments applied for multiple comparisons. For COAST scores, the change in score from baseline to the final event was calculated for each dog, and group differences were assessed using a Wilcoxon rank-sum test. A generalized linear mixed model was used to evaluate individual patient success as a binary outcome. Fixed effects included the treatment group and the event. Additional generalized linear mixed models were fitted separately within each group to assess the effects of the event and the %improvement on DJA, with NSAID use included as a covariate. For the OA score and the %improvement on DJA, which were only assessed at a single time point, two-sample t-tests were used to compare means between groups. A correlation analysis was also performed to evaluate the relationship between the OA score and the %improvement on DJA. To assess differences in the proportion of dogs that restarted NSAIDs between groups, both Chi-squared and Fisher’s exact tests were used. The *p*-value from Fisher’s exact test is reported in the manuscript due to the small sample size. The residuals from the model evaluating Total Daily Activity Count were right-skewed; therefore, a log transformation was applied to meet assumptions of normality.

## Results

Thirty-five dogs qualified for enrollment in the study. Of those, 17 were spayed females, 17 were neutered males, and one was an intact male. Weight ranged from 10.1 kg to 56.9 kg (mean = 31.6 SE ± 11.8 kg), and age ranged from 1.5 to 14 years old (mean = 8.0 years SE ± 3.3). Supplementary Table 1 lists patient signalment, the most clinically affected elbow, NSAID administration, group allocation, and possible data exclusions. One dog was excluded due to receiving an overdose of NSAIDs at the time of enrollment, and another dog was excluded due to developing iatrogenic Addison’s disease after Visit 3 (unrelated to the study). Thirty-three patients were included in at least one partial analysis. Eleven patients had partial exclusion of data for various reasons, summarized in Supplementary Table 1. When comparing the treatment groups at baseline, there was insufficient evidence of a difference in the OA score or the %improvement on the DJA between groups. There was also insufficient evidence of a difference in %BWD, SOS, CSOM ACT, CSOM BEHAV, CBPI PIS, CBPI QOL, COAST, age, or weight between groups. However, there was evidence of a statistically significant difference in CBPI PSS between groups (*p* = 0.008). This is outlined in Table 3.

**Table 3:** Baseline comparisons between treatment groups.

Comparisons	TA + CEHM group	TA group	*p* value
*n*	17	16	
%BWD (mean (SD))	27.30 (2.74)	26.23 (4.28)	0.397
SOS (mean (SD))	11.65 (3.14)	11.75 (2.65)	0.92
CSOM. ACT(MEAN) (mean (SD))	3.13 (0.62)	3.25 (0.69)	0.589
CSOM. BEHAV(MEAN) (mean (SD))	2.67 (0.70)	2.35 (0.82)	0.248
CBPI. PSS (mean (SD))	5.94 (1.23)	4.64 (1.41)	0.008*
CBPI. PIS (mean (SD))	6.02 (1.78)	5.45 (1.88)	0.376
CBPI. QOL (median [IQR])	3.00 [3.00, 3.00]	3.00 [2.00, 3.00]	0.598
COAST (median [IQR])	3.00 [3.00, 3.00]	3.00 [3.00, 3.00]	0.729
OA score (mean (SD))	61.12 (31.75)	40.75 (26.05)	0.054
Age (years) (mean (SD))	8.06 (3.51)	7.53 (3.05)	0.649
Weight (kgs) (mean (SD))	32.40 (12.60)	31.73 (11.52)	0.874

SD, standard deviation; IQR, interquartile range; %BWD, body weight distribution; SOS, subjective orthopedic scoring; CBPI, Canine Brief Pain Inventory; PSS, pain severity score; PIS, pain interference score; QOL, quality of life; CSOM, client subjective outcome measure; ACT, activity; BEHAV, behavior; COAST, canine osteoarthritis staging tool (*) indicates statistically significant difference between groups (*p* value < 0.05).

A summary of the results obtained from the linear models of the NSAID-adjusted data, for all outcome measures and time points, is reported in Supplementary Table 2. Insufficient evidence was found to conclude that the treatment group was associated with any response variable. Therefore, no pairwise comparisons were estimated to compare treatment groups. Using NSAID-adjusted data, the differences in the model-based mean predicted values between treatment groups were estimated at each post-injection time point for the key outcome measures. The results are presented as mean differences with 95% confidence intervals (CIs). The %BWD results are as follows: 0 (95% CI: −2.61 to 2.62), 0.49 (95% CI: −2.14 to 3.13), 0.7 (95% CI: −1.98 to 3.37), and 1.22 (95% CI: −1.62 to 4.07) for visits 4, 5, 6, and 7, respectively. The results for CBPI PSS include: 0.8 (95% CI: −0.42 to 2.02), 0.78 (95% CI: −0.45 to 2), −0.01 (95% CI: −1.26 to 1.24), and −0.05 (95% CI: −1.38 to 1.29) for visits 4, 5, 6, and 7, respectively. Results for the CBPI PIS include: 0.41 (95% CI: −1.15 to 1.98), 0.17 (95% CI: −1.4 to 1.74), −0.76 (95% CI: −2.35 to 0.83), and −0.27 (95% CI: −1.93 to 1.39) for visits 4, 5, 6, and 7, respectively.

Several subjective outcome measures improved for both groups (see Supplementary Table 2). Subjective outcome measures that had sufficient evidence of a difference from baseline in the TA + CEHM group included: SOS at visit 4, CSOM BEHAV at visits 4, 5, 6, and 7, CBPI PSS at visits 4, 5, 6, and 7, and CBPI PIS at visits 4, 5, 6, and 7. Subjective outcome measures that had sufficient evidence of a difference from baseline in the TA group included: CSOM ACT at visits 4, 6, and 7, CSOM BEHAV at visits 4 and 6, CBPI PSS at visits 4, 5, and 7, and CBPI PIS at visits 4 and 5.

There was no significant difference in individual patient success between groups (Table 4). Supplementary Table 3 outlines which patients reached success at each time point, the total TA dose, and whether they restarted NSAIDs. The greatest success rate (29%) was identified in the TA + CEHM group at Visit 6, with a success rate of 21% in the TA group at the same time point (32). Of the dogs that received at least a 0.2 mg/kg total dose of TA (regardless of treatment group), 3/7 (43%) achieved individual patient success; of the dogs that received less than a 0.2 mg/kg total dose of TA, 5/26 (19%) achieved individual patient success. There was not enough evidence to conclude that OA scoring is correlated with %improvement on DJA (Pearson Correlation = 0.001) (see Table 5). Accelerometry data appeared unaffected in both groups.

**Table 4:** Overall individual patient success compared to baseline at each visit between groups.

Visit #	Success TA + CEHM	Success TA
Visit 4	(0/17) 0%	(1/16) 6%
Visit 5	(2/16) 13%	(1/16) 6%
Visit 6	(5/17) 29%	(3/14) 21%
Visit 7	(2/15) 20%	(1/10) 10%

**Table 5:** Percent improvement in DJA and OA scores between treatment groups.

Outcomes	Group	Mean	Standard error
%improvement on DJA	TA + CEHM	15.53	6.32
%improvement on DJA	TA	26.11	31.70
OA score	TA + CEHM	61.12	31.75
OA score	TA	40.75	26.05

Eighty-two percent (14/17) of dogs in the TA + CEHM group were documented to have restarted NSAIDs compared to 56% (9/16) of dogs in the TA group. There was insufficient evidence to show a difference in restarting NSAIDs between groups (*p* = 0.14). All but one patient in the TA group complied with NSAID washout instructions. This patient took consistent NSAIDs throughout the trial and was included among the 9/16 dogs that restarted NSAIDs.

Owner-reported adverse events of TA + CEHM included transient soreness ranging from mild to moderate, lethargy, and ataxia. Mild transient soreness was reported in the TA group, but no other adverse events were reported. This is summarized in Table 6. All adverse events experienced during the study period resolved without treatment.

**Table 6:** Adverse events.

Adverse event	TA + CEHM (*n* = 17)	TA (*n* = 16)
Mild transient soreness	(3/17) 18%	(2/16) 13%
Moderate transient soreness	(2/17) 12%	(0/16) 0%
Lethargy	(1/17) 6%	(0/16) 0%
Ataxia	(1/17) 6%	(0/16) 0%

Adverse events were based on a post-injection survey sent to the owners 3 days after injection.

## Discussion

This double-blind, positive-controlled, clinical trial was conducted to gather preliminary evidence evaluating the efficacy and tolerability of a novel CEHM product in client-owned dogs with bilateral elbow OA. While several subjective outcome measures differed from baseline in both groups, neither objective outcome measure (OGA/accelerometry) showed a statistically significant difference between groups or compared to baseline. Given that this study used a positive control, both owners and veterinarians were aware that all patients received treatment (TA + CEHM or TA alone). Consequently, our subjective outcome measures were likely affected by the caregiver placebo effect (33). In dogs with lameness related to OA, a caregiver placebo effect (i.e., perceived improvement without active treatment) of ~45–57% is reported (33). As such, the observed improvement in subjective outcome measurements from baseline was expected and cannot be interpreted as treatment success. A difference between treatment groups would have to be observed to conclude the superiority of one treatment over the other.

Although no evidence was found to support a significant difference between groups, a higher proportion of individual patient success was observed in the TA + CEHM group at visits 5, 6, and 7. This may have been due to a higher (although statistically insignificant) proportion of patients in the TA + CEHM group resuming NSAIDs, or it may have been due to subtle improvements in the TA + CEHM group compared to the TA group. Additionally, there was a difference in CBPI PSS between groups at baseline, with the TA + CEHM groups having a higher pain score. There was no difference detected between groups at any other time point. This could be interpreted as the superiority of the TA + CEHM group; however, the higher proportion of patients resuming NSAIDs may also explain this finding. Conversely, there was a higher proportion of adverse events in the TA + CEHM group. These findings clearly indicate that further studies with larger sample sizes are needed. Based on the available data, it is not possible to conclude whether one treatment group is superior to the other or is associated with more adverse events.

All patients were required to have consistent NSAID administration (for at least 4 weeks) prior to enrollment in the study in order to create consistency across groups. Additionally, all but one patient discontinued NSAIDs 5 days prior to injection (due to the owner’s non-compliance). Data collected at the injection visit were used as a baseline. It is likely that data collected from this timepoint were influenced by NSAID withdrawal. However, since patients in both groups were required to discontinue NSAIDs, this did not influence the comparison of outcome measures between groups. NSAIDs were discontinued due to potential drug interaction from the systemic effects of TA (32). For ethical reasons, and so that re-instatement could be used as an outcome measure, patients were allowed to resume NSAIDs after injection if deemed necessary for pain management. This is expected to have affected the results, as a higher proportion of patients in the TA + CEHM group resumed NSAID administration. Therefore, NSAIDs were included as a covariate in the statistical model. Disallowing NSAIDs for the duration of the study would have provided a clearer interpretation of the results, but this approach was not deemed ethical.

Adverse events reported in the TA + CEHM group in the present study included mild-to-moderate transient soreness, lethargy, and ataxia. In the TA group, the only reported adverse event was mild transient soreness. These events were documented via a survey sent to owners 3 days post joint injections. All adverse events resolved without treatment. Transient soreness was suspected based on owner reports of pain and lameness that resolved without treatment; however, a clinical workup was not performed to confirm this diagnosis. In the TA + CEHM group, more dogs experienced transient soreness compared to the TA group. The rate of transient soreness in the TA + CEHM group was higher than the expected rate for other intra-articular injections. The existing literature has reported an expected rate of transient soreness of 18.4% with intra-articular injection. According to past studies, in the stifle and tarsus, larger injectate volumes are more likely to be associated with transient soreness (34). It is possible that the larger injectate volume in the TA + CEHM group contributed to this higher rate. Patients experiencing lethargy and ataxia were requested to return for a recheck; however, this was declined by the owners in both cases. Given the rapid resolution of clinical symptoms, it is difficult to determine whether these adverse events were due to sedation or the injected product. Because clinical evaluations were optional and not required, reporting bias or misclassification of adverse events is possible.

This study had several limitations. Due to financial reasons, the number of cases enrolled was below the determined sample size for the majority of outcome measures, including OGA and accelerometry (30). The sample size was only met for the CBPI PSS. To account for these shortcomings, individual patient success criteria were used to evaluate success at the patient level. While these criteria were based on previous literature where available, the determination that four of the five success criteria had to be met for individual patient success was arbitrary. The significance of treatment success has traditionally been determined by comparing the means of outcome measures between groups. Predefining individual success criteria is an alternative approach that can be used to determine success on an individual patient basis. This approach relies on minimal clinically important differences (MCID) to determine the difference needed for a given outcome measure to be considered successful (35). MCID has been established in the literature for some outcome measures (i.e., OGA, CBPI, and accelerometry) (27, 29–31, 33, 36). However, cut-offs for other outcome measures (i.e., SOS) were determined arbitrarily due to a lack of literature. Further research is needed to determine individual patient success criteria when using various outcome measurements.

DJA was used to confirm the elbow joint as the source of lameness in the study patients. This approach was used to increase confidence in the localization of the dog’s pain (e.g., to confirm that lameness was in fact due to elbow arthritis rather than disease in other locations). The %improvement qualifying as a positive DJA used in this study was 5%, based on the established MCID for OGA, which varies between 3 and 5% (29, 33, 36). However, the degree of improvement on OGA that verifies the injected joint as the source of lameness has not been determined to date. Previous research, using subjective lameness scoring, has used an improvement of at least two grades (based on an 11-point Likert scale) as the cut-off to confirm a positive response (26). The 5% cutoff point chosen in this study may have allowed for the enrollment of cases with false-positive results. In dogs, DJA is typically performed under sedation to maintain a safe and sterile arthrocentesis technique. The use of sedatives, especially opioids, may affect the outcome of gait analysis post injection (37). In this study, the use of opioids was avoided; however, some patients required them for adequate sedation. Finally, in cases where it was deemed in the patient’s best interest, diagnostics were performed under the same sedation event. This prolonged sedation may also have influenced the outcome of OGA. Despite these limitations, the authors believe that the utilization of DJA prior to enrollment provides an opportunity for more accurate patient selection in future studies.

Overall, this study population had a high degree of OA. It is possible that dogs with late-stage OA may have less potential to respond to intra-articular therapy, due to the more severe subchondral bone pathology present in these cases (38). In late-stage OA, it is more likely to see significant damage to the bone surrounding joints. Although cartilage is aneural, the soft tissues and subchondral bone surrounding joints are robustly innervated by sensory nerves. When subchondral bone is damaged, bone marrow lesions and sclerosis may lead to damage to the nociceptive nerve fibers (39, 40). Due to the innervation of the subchondral bone arising from the medullary cavity, it has been hypothesized that intra-articular injection of DJA may be ineffective in desensitizing this type of pain in horses (41). Consequently, intra-articular treatment may not be an effective treatment for subchondral bone pain. In general, elbow dysplasia has a variable disease course and response to treatment. In this study, CT was not used consistently, which made it impossible to classify patients by the type of elbow dysplasia (42). These factors may have led to limitations in the response to intra-articular treatment.

In the present study, enrollment criteria required dogs to have bilateral elbow OA, and both elbows were treated. This protocol was chosen based on the high prevalence of bilateral involvement in canine elbow dysplasia to ensure a more comprehensive therapeutic approach for affected patients (43). This approach may result in greater improvement in overall comfort and activity. However, it is possible that this bilateral treatment approach may have masked the perception of improvement for OGA, since both joints may improve. In this case, an improvement in activity data would be expected. Given the lack of improvement in accelerometry data, a lack of treatment effect is suspected. However, because of the limitations of both the outcome measures and the study sample size, it is difficult to draw this conclusion.

The use of corticosteroids as an intra-articular treatment for OA pain has been well documented in dogs and humans (10, 44). However, the TA dose utilized was not representative of current recommendations for all dogs. At the time of the study design, the manufacturer recommended using TA in combination with their product; as such, it was chosen for the control group at the same dose. The lack of improvement in objective outcome measures in both groups may be due to the dose used or because of the true inefficacy of the treatment. The dose of TA used in this study ranged from 0.07 to 0.2 mg/kg/joint, depending on where the dog fell in the weight range. A higher total dose of at least 0.2 mg/kg is frequently used in clinical practice for intra-articular injections in dogs (32). Of the dogs that received a total dose of at least 0.2 mg/kg of TA (regardless of treatment group), 3/7 (43%) achieved individual patient success, whereas of the dogs that received a total dose of less than 0.2 mg/kg of TA, 5/26 (19%) achieved individual patient success. Although this may have contributed to the lack of efficacy in objective outcome measures, it is difficult to draw conclusions due to the small sample size.

## Conclusion

The present study found insufficient evidence to conclude that the TA + CEHM group was superior to the TA group based on any of the outcome measures. Adverse events reported during the study period resolved without treatment. Given the lack of difference between groups, further research, with larger sample sizes, is needed to justify the use of these products in dogs for the treatment of elbow OA.

## Data Availability

The raw data supporting the conclusions of this article will be made available by the authors, without undue reservation.
