# Supplementary material for: Evaluation of intra-articular injection of collagen-elastin hydrogel microparticles for managing osteoarthritis-associated elbow pain in dogs: a double-blind, positive-controlled clinical trial
Source: Front Vet Sci. 2026 Feb 11;13:1742766. doi: 10.3389/fvets.2026.1742766 (PMC12932243; doi:10.3389/fvets.2026.1742766)
Supplement: Supplementary file 1 [file Table_1.DOCX]

**Supplementary Table 1: Patient demographic and clinical information, group allocation, NSAID administration, and reasons for possible data exclusion.**

| **Patient** | **Age (years)** | **Sex** | **Breed** | **Most Affected Elbow** | **Group Allocation** | **Restart NSAIDs (yes/no)** | **Data exclusion** |
| --- | --- | --- | --- | --- | --- | --- | --- |
| 1 | 11 | FS | Mixed breed | R Elbow | TA + CEHM | YES | -- |
| 2 | 3 | MN | Malamute | R Elbow | TA + CEHM | NO | -- |
| 3 | 8 | MN | Labrador Retriever | L Elbow | TA | NO | Data excluded at visit 7 due to hemoabdomen |
| 4 | 5 | FS | Labrador Retriever | L Elbow | TA + CEHM | YES | -- |
| 5 | 8 | MN | Mixed breed | L Elbow | TA | NO | -- |
| 6 | 6 | MN | Mixed breed | R Elbow | TA + CEHM | YES | -- |
| 7 | 1.5 | MN | Mixed breed | L Elbow | TA | YES | Excluded COAST data from visit 7 due to missing data |
| 8 | 9 | MN | Mixed breed | R Elbow | TA | NO | All data excluded at visits 6-7 due to patient withdrawing from the study |
| 9 | 7 | FS | Mixed breed | R Elbow | TA | NO | All data excluded at visit 7 due to patient being euthanized for unrelated reasons |
| 10 | 2 | MI | Labrador Retriever | L Elbow | TA + CEHM | YES | -- |
| 11 | 10 | FS | Mixed breed | L Elbow | TA + CEHM | YES | -- |
| 12 | 8 | FS | Mixed breed | R Elbow | TA | YES | -- |
| 13 | 10 | MN | Mixed breed | L Elbow | TA + CEHM | YES | -- |
| 14 | 4 | MN | Labrador Retriever | L Elbow | TA | YES | Excluded COAST data from visit 7 due to missing data |
| 15 | 5 | MN | Rottweiler | L Elbow | TA | NO | -- |
| 16 | 10 | FS | Australian Cattle Dog | L Elbow | TA + CEHM | YES | -- |
| 17 | 11 | FS | Goldendoodle | R Elbow | TA + CEHM | YES | -- |
| 18 | 9 | FS | Mixed Breed | R Elbow | TA | YES | -- |
| 19 | 7 | MN | Labrador Retriever | R Elbow | TA | YES | All data excluded at visits 7 due to patient withdrawing from the study |
| 20 | 4 | MN | Mixed Breed | L Elbow | TA | NO | All data excluded at visits 7 due to patient withdrawing from the study |
| 21 | 4 | MN | Mixed Breed | R Elbow | TA + CEHM | NO | -- |
| 22 | 10 | FS | Mixed Breed | R Elbow | TA + CEHM | YES | -- |
| 23 | 5 | FS | Golden Retriever | R Elbow | TA | NO | -- |
| 24 | 11 | FS | Golden Retriever | R Elbow | TA + CEHM | YES | -- |
| 25 | 11 | FS | Mixed breed | R Elbow | TA | YES | All data excluded at visits 6-7 due to patient withdrawing from the study |
| 26 | 11 | FS | Labrador Retriever | L Elbow | TA | YES | -- |
| 27 | 6 | MN | Labrador Retriever | R Elbow | TA + CEHM | YES | -- |
| 28 | 6 | MN | Mixed Breed | L Elbow | TA + CEHM | NO | -- |
| 29 | 14 | MN | Mixed Breed | L Elbow | TA + CEHM | YES | All data excluded visits 6-7 due to patient receiving extracorporeal shockwave treatment to left elbow |
| 30 | 12 | FS | Border Collie | R Elbow | TA + CEHM | YES | All data excluded at visit 7 due to patient receiving intra-articular injection into right elbow |
| 31 | 10 | FS | Shetland Sheepdog | R Elbow | TA | YES | -- |
| 32 | 6 | MN | German Shepherd | L Elbow | TA + CEHM | YES | All data excluded at visit 5 due to patient missing study appointment |
| 33 | 13 | MN | Pug x Beagle | L Elbow | TA | YES | -- |

This table refers to the age and most affected elbow at the time of enrollment. Restarting NSAIDs indicates if the patients restarted NSAIDs at any point during the trial, after injection. L elbow = left elbow; R elbow = right elbow; MN = male neutered; FS = female spayed; MI = male intact.

**Supplementary Table 2: Results for all outcome measures across study visits by group:**

| **Outcome Measure** | **Group Allocation** | **Mean (SE) at baseline (visit 3)** | **Mean (SE) at visit 4** | **Mean (SE) at visit 5** | **Mean (SE) at visit 6** | **Mean (SE) at visit 7** |
| --- | --- | --- | --- | --- | --- | --- |
| ↓ACTICAL.SED (minutes) | TA + CEHM | 1149.13 (16.62) | 1148.67 (16.35) | 1149.35 (16.34) | 1156.91 (16.49) | 1157.68 (16.63) |
| ↓ACTICAL.SED (minutes) | TA | 1186.07 (17.23) | 1187.11 (16.93) | 1187.60 (16.86) | 1196.65 (17.22) | 1191.91 (17.76) |
| ACTICAL.LIGHT (minutes) | TA + CEHM | 186.45 (10.49) | 188.53 (10.30) | 185.49 (10.29) | 180.25 (10.40) | 181.58 (10.49) |
| ACTICAL.LIGHT (minutes) | TA | 168.21 (10.87) | 168.64 (10.67) | 165.88 (10.62) | 164.18 (10.87) | 167.92 (11.23) |
| ↑ACTICAL.MOD (minutes) | TA + CEHM | 104.21 (8.94) | 102.18 (8.81) | 104.63 (8.80) | 102.60 (8.88) | 100.55 (8.95) |
| ↑ACTICAL.MOD (minutes) | TA | 85.47 (9.27) | 83.91 (9.11) | 86.24 (9.08) | 79.04 (9.26) | 79.96 (9.53) |
| ↑TOTAL ACTIVITY COUNT Log(minutes) | TA + CEHM | 11.39 (0.10) | 11.43 (0.10) | 11.42 (0.10) | 11.42 (0.10) | 11.39 (0.10) |
| ↑TOTAL ACTIVITY COUNT Log(minutes) | TA | 11.24 (0.11) | 11.25 (0.11) | 11.26 (0.11) | 11.15 (0.11) | 11.18 (0.11) |
| ↑%BWD | TA + CEHM | 26.84 (0.97) | 27.20 (0.92) | 27.28 (0.92) | 27.85 (0.93) | 28.03 (0.95) |
| ↑%BWD | TA | 25.83 (0.98) | 27.20 (0.93) | 26.79 (0.93) | 27.16 (0.96) | 26.81 (1.07) |
| ↓SOS (0-24) | TA + CEHM | 12.33 (0.81) | 10.40 (0.80)* | 10.82 (0.78) | 11.04 (0.79) | 11.94 (0.80) |
| ↓SOS (0-24) | TA | 12.35 (0.82) | 10.96 (0.80) | 11.05 (0.80) | 10.89 (0.81) | 11.10 (0.86) |
| ↓CSOM.ACT (1-5) | TA + CEHM | 3.22 (0.22) | 2.73 (0.21) | 2.95 (0.21) | 2.82 (0.21) | 2.98 (0.22) |
| ↓CSOM.ACT (1-5) | TA | 3.33 (0.22) | 2.75 (0.21)* | 2.87 (0.21) | 2.75 (0.22)* | 2.60 (0.24)* |
| ↑CSOM.BEHAV (1-5) | TA + CEHM | 2.50 (0.21) | 3.22 (0.20)* | 3.18 (0.20)* | 3.30 (0.20)* | 3.31 (0.20)* |
| ↑CSOM.BEHAV (1-5) | TA | 2.21 (0.21) | 2.75 (0.20)* | 2.63 (0.20) | 2.69 (0.21)* | 2.66 (0.22) |
| ↓CBPI.PSS (0-10) | TA + CEHM | 6.60 (0.46) | 4.42 (0.43)* | 4.72 (0.43)* | 4.08 (0.44)* | 3.78 (0.44)* |
| ↓CBPI.PSS (0-10) | TA | 5.22 (0.46) | 3.61 (0.43)* | 3.95 (0.43)* | 4.08 (0.45) | 3.83 (0.50)* |
| ↓CBPI.PIS (0-10) | TA + CEHM | 6.50 (0.57) | 4.72 (0.55)* | 4.88 (0.55)* | 4.14 (0.55)* | 4.37 (0.56)* |
| ↓CBPI.PIS (0-10) | TA | 5.87 (0.58) | 4.30 (0.55)* | 4.71 (0.55)* | 4.90 (0.57) | 4.64 (0.61) |

This table provides a summary of results obtained by the linear models (adjusting for NSAID use) including model based means and standard errors. SE, standard error; BWD, body weight distribution; SED, sedentary; MOD, moderate; SOS, subjective orthopedic scoring; CBPI, Canine Brief Pain Inventory; PSS, pain severity score; PIS, pain interference score; CSOM, client subjective outcome measure; ACT, activity; BEHAV, behavior. Direction of arrow next to listed outcomes denotes the direction of value which indicates a more favorable response (i.e. ↓ represents that a lower score equates to clinical improvement and vice versa). (*) indicates statistically significant difference from baseline (p value < 0.05).

**Supplementary Table 3: Overall individual patient success at each visit and TA dose received**

| **Patient #** | **Treatment Group** | **Visit #** | **Overall Individual Patient Success** | **TA total dose (mg/kg)** |
| --- | --- | --- | --- | --- |
| 2 | TA + CEHM | 4 | NO | 0.15 |
| 2 | TA + CEHM | 5 | YES |  |
| 2 | TA + CEHM | 6 | YES |  |
| 2 | TA + CEHM | 7 | YES |  |
| 23 | TA | 4 | NO | 0.16 |
| 23 | TA | 5 | YES |  |
| 23 | TA | 6 | YES |  |
| 23 | TA | 7 | NO |  |
| 28 | TA + CEHM | 4 | NO | 0.21 |
| 28 | TA + CEHM | 5 | NO |  |
| 28 | TA + CEHM | 6 | YES |  |
| 28 | TA + CEHM | 7 | YES |  |
| 18 | TA | 4 | YES* | 0.24 |
| 18 | TA | 5 | NO* |  |
| 18 | TA | 6 | YES* |  |
| 18 | TA | 7 | YES* |  |
| 4 | TA + CEHM | 4 | NO* | 0.18 |
| 4 | TA + CEHM | 5 | NO* |  |
| 4 | TA + CEHM | 6 | YES* |  |
| 4 | TA + CEHM | 7 | YES* |  |
| 10 | TA + CEHM | 4 | NO* | 0.14 |
| 10 | TA + CEHM | 5 | YES* |  |
| 10 | TA + CEHM | 6 | YES* |  |
| 10 | TA + CEHM | 7 | NO* |  |
| 7 | TA | 4 | NO* | 0.16 |
| 7 | TA | 5 | NO* |  |
| 7 | TA | 6 | YES* |  |
| 7 | TA | 7 | NO* |  |
| 11 | TA + CEHM | 4 | NO* | 0.36 |
| 11 | TA + CEHM | 5 | NO* |  |
| 11 | TA + CEHM | 6 | YES* |  |
| 11 | TA + CEHM | 7 | NO* |  |
| 3 | TA | 4 | NO | 0.15 |
| 3 | TA | 5 | NO |  |
| 3 | TA | 6 | NO |  |
| 3 | TA | 7 |  |  |
| 5 | TA | 4 | NO | 0.17 |
| 5 | TA | 5 | NO |  |
| 5 | TA | 6 | NO |  |
| 5 | TA | 7 | NO |  |
| 8 | TA | 4 | NO | 0.14 |
| 8 | TA | 5 | NO |  |
| 8 | TA | 6 |  |  |
| 8 | TA | 7 |  |  |
| 9 | TA | 4 | NO | 0.17 |
| 9 | TA | 5 | NO |  |
| 9 | TA | 6 | NO |  |
| 9 | TA | 7 |  |  |
| 15 | TA | 4 | NO | 0.17 |
| 15 | TA | 5 | NO |  |
| 15 | TA | 6 | NO |  |
| 15 | TA | 7 | NO |  |
| 20 | TA | 4 | NO | 0.19 |
| 20 | TA | 5 | NO |  |
| 20 | TA | 6 | NO |  |
| 20 | TA | 7 |  |  |
| 21 | TA + CEHM | 4 | NO | 0.17 |
| 21 | TA + CEHM | 5 | NO |  |
| 21 | TA + CEHM | 6 | NO |  |
| 21 | TA + CEHM | 7 | NO |  |
| 25 | TA | 4 | NO* | 0.17 |
| 25 | TA | 5 | NO* |  |
| 25 | TA | 6 |  |  |
| 25 | TA | 7 |  |  |
| 19 | TA | 4 | NO* | 0.18 |
| 19 | TA | 5 | NO* |  |
| 19 | TA | 6 | NO* |  |
| 19 | TA | 7 |  |  |
| 29 | TA + CEHM | 4 | NO* | 0.19 |
| 29 | TA + CEHM | 5 | NO* |  |
| 29 | TA + CEHM | 6 | NO* |  |
| 29 | TA + CEHM | 7 |  |  |
| 30 | TA + CEHM | 4 | NO* | 0.16 |
| 30 | TA + CEHM | 5 | NO* |  |
| 30 | TA + CEHM | 6 | NO* |  |
| 30 | TA + CEHM | 7 |  |  |
| 32 | TA + CEHM | 4 | NO* | 0.13 |
| 32 | TA + CEHM | 5 |  |  |
| 32 | TA + CEHM | 6 | NO* |  |
| 32 | TA + CEHM | 7 | NO* |  |
| 1 | TA + CEHM | 4 | NO* | 0.19 |
| 1 | TA + CEHM | 5 | NO* |  |
| 1 | TA + CEHM | 6 | NO* |  |
| 1 | TA + CEHM | 7 | NO* |  |
| 6 | TA + CEHM | 4 | NO* | 0.2 |
| 6 | TA + CEHM | 5 | NO* |  |
| 6 | TA + CEHM | 6 | NO* |  |
| 6 | TA + CEHM | 7 | NO* |  |
| 12 | TA | 4 | NO* | 0.11 |
| 12 | TA | 5 | NO* |  |
| 12 | TA | 6 | NO* |  |
| 12 | TA | 7 | NO* |  |
| 13 | TA + CEHM | 4 | NO* | 0.18 |
| 13 | TA + CEHM | 5 | NO* |  |
| 13 | TA + CEHM | 6 | NO* |  |
| 13 | TA + CEHM | 7 | NO* |  |
| 14 | TA | 4 | NO* | 0.21 |
| 14 | TA | 5 | NO* |  |
| 14 | TA | 6 | NO* |  |
| 14 | TA | 7 | NO* |  |
| 16 | TA + CEHM | 4 | NO* | 0.18 |
| 16 | TA + CEHM | 5 | NO* |  |
| 16 | TA + CEHM | 6 | NO* |  |
| 16 | TA + CEHM | 7 | NO* |  |
| 17 | TA + CEHM | 4 | NO* | 0.16 |
| 17 | TA + CEHM | 5 | NO* |  |
| 17 | TA + CEHM | 6 | NO* |  |
| 17 | TA + CEHM | 7 | NO* |  |
| 22 | TA + CEHM | 4 | NO* | 0.16 |
| 22 | TA + CEHM | 5 | NO* |  |
| 22 | TA + CEHM | 6 | NO* |  |
| 22 | TA + CEHM | 7 | NO* |  |
| 24 | TA + CEHM | 4 | NO* | 0.12 |
| 24 | TA + CEHM | 5 | NO* |  |
| 24 | TA + CEHM | 6 | NO* |  |
| 24 | TA + CEHM | 7 | NO* |  |
| 26 | TA | 4 | NO* | 0.18 |
| 26 | TA | 5 | NO* |  |
| 26 | TA | 6 | NO* |  |
| 26 | TA | 7 | NO* |  |
| 27 | TA + CEHM | 4 | NO* | 0.15 |
| 27 | TA + CEHM | 5 | NO* |  |
| 27 | TA + CEHM | 6 | NO* |  |
| 27 | TA + CEHM | 7 | NO* |  |
| 31 | TA | 4 | NO* | 0.28 |
| 31 | TA | 5 | NO* |  |
| 31 | TA | 6 | NO* |  |
| 31 | TA | 7 | NO* |  |
| 33 | TA | 4 | NO* | 0.27 |
| 33 | TA | 5 | NO* |  |
| 33 | TA | 6 | NO* |  |
| 33 | TA | 7 | NO* |  |

(*) indicates that the patient was receiving NSAIDs at that visit. The table is organized in descending order so that patients who had the most instances of “overall individual patient success” are at the top. Secondly, patients were organized in ascending order with the least amount of NSAID use at the top.
